# Increased hepatic Th2 and Treg subsets are associated with biliary fibrosis in different strains of mice caused by *Clonorchis sinensis*

**DOI:** 10.1371/journal.pone.0171005

**Published:** 2017-02-02

**Authors:** Bei-Bei Zhang, Chao Yan, Fan Fang, Ying Du, Rui Ma, Xiang-Yang Li, Qian Yu, Di Meng, Ren-Xian Tang, Kui-Yang Zheng

**Affiliations:** 1 Jiangsu Key Laboratory of Immunity and Metabolism, Department of Pathogenic Biology and Immunology, Laboratory of Infection and Immunity, Xuzhou Medical University, Xuzhou, Jiangsu Province, People's Republic of China; 2 Departments of Pathology, the Affiliated Hospital of Xuzhou Medical University, Xuzhou, China; Centro de Investigacion y de Estudios Avanzados del Instituto Politecnico Nacional, MEXICO

## Abstract

Previous studies showed that CD4^+^T cells responses might be involved in the process of biliary fibrosis. However, the underlying mechanism resulting in biliary fibrosis caused by *Clonorchis sinensis* remains not yet fully elucidated. The objectives of the present study were to investigate the different profiles of hepatic CD4^+^T cell subsets (Th1, Th2, Th17 and Treg cells) and their possible roles in the biliary fibrosis of different strains of mice (C57BL/6, BALB/c and FVB mice) induced by *C*. *sinensis* infection. C57BL/6, BALB/c and FVB mice were orally gavaged with 45 metacercariae. All mice were sacrificed on 28 days post infection in deep anesthesia conditions. The leukocytes in the liver were separated to examine CD4^+^T cell subsets by flow cytometry and the left lobe of liver was used to observe pathological changes, collagen depositions and the concentrations of hydroxyproline. The most serious cystic and fibrotic changes appeared in FVB infected mice indicated by gross observation, Masson’s trichrome staining and hydroxyproline content detection. In contrast to C57BL/6 infected mice, diffuse nodules and more intensive fibrosis were observed in the BALB/c infected mice. No differences of the hepatic Th1 subset and Th17 subset were found among the three strains, but the hepatic Th2 and Treg cells and their relative cytokines were dramatically increased in the BALB/c and FVB infected groups compared with the C57BL/6 infected group (*P*<0.01). Importantly, increased Th2 subset and Treg subset all positively correlated with hydroxyproline contents (*P*<0.01). This result for the first time implied that the increased hepatic Th2 and Treg cell subsets were likely to play potential roles in the formation of biliary fibrosis in *C*. *sinensis*-infected mice.

## Introduction

Clonorchiasis is one of the most common food-borne trematodiases caused by *Clonorchis sinensis* infection that is prevalent in Eastern Asia including China, Korea, Vietnam and eastern Russia [1]. After ingestion of the undercooked fish containing *C*. *sinensis* metacercariae, the encysts are released owing to the stimulation of gastric juice and the cyst wall is digested by trypsin in the duodenum. Juvenile worms colonize in the bile ducts of hosts and lead to the damage to biliary epithelium, resulting in cholangitis, cholelithiasis, biliary fibrosis, and the cholangiocarcinoma [2–8].

Outcomes of parasitic infection is determined by a large number of factors, like host genetic variation, different immunological backgrounds and nutritional status [9–12]. Thus, these factors may probably not only influence the liver pathological lesions triggered by invasion of *C*. *sinensis*, but also the growth and clearance of this parasite. For example, previous studies reported that FVB mice are prone to form cysts, while BALB/c mice develops more extensive biliary fibrosis when they were administrated the same amount of *C*. *sinensis* metacercariae. There are also differences in other hepatic pathological changes such as inflammatory cells infiltration, fibrotic proliferation and biliary hyperplasia between these two strains [13]. However, the underlying mechanism resulting in these differences remains not yet fully elucidated.

Originally, CD4^+^T-helper 2 cells immune responses are responsible for the main evolution driving by the helminth infection [14]. However, with the further understandings and definitions of CD4^+^T cells, new T-helper cell subsets such as Th17 cells and regulatory T cells were also found as it proved that these cell subsets play crucial roles in helminth infection [15–17]. It is clear that the differentiation of these four subsets including Th1, Th2, Th17 and Treg requires specific cytokine milieu [18, 19]. These four populations secreted distinct profiles of functional cytokines that may play vital roles in protection against the parasite invasion, in regulation of host immune responses and histopathological lesions, as well as in expulsion of parasites [20]. For instance, Th1 and Th2 cells are involved in the clearance of intracellular pathogens and helminthic parasites, respectively [21]. Th1 cells can inhibit the liver fibrosis because the secreted IFN-γ has the suppressive effect on the activation of hepatic stellate cells. But Th2 cytokines such as IL-4, IL-5 and IL-13 promote the development of fibrosis by inducing activation of hepatic stellate cells [22]. It has also been reported that Th17 induced the formation of liver granuloma and fibrosis via selectively producing IL-17 cytokine in schistosomiasis while Treg was reported to play roles in down regulating such hepatic pathological injuries [23,24]. However, there have been limited data implicating the pattern of CD4^+^T cell subsets appeared during the *C*. *sinensis* infection. Our previous study indicated that Th1 immune response, which appeared during the acute phase, would shift to Th2 immune response with the infection developing [25]. Apart from this, there was an imbalance between Treg and Th17 cells, which might contribute to the hepatic fibrosis in *C*. *sinensis* infected BALB/c mice [26]. Nevertheless, little is known regarding the amounts of liver CD4^+^T cell subsets and its relationship with biliary fibrosis in different strains of *C*. *sinensis*-infected mice. Therefore, in the present study, C57BL/6, BALB/c and FVB mice infected with *C*. *sinensis* were employed to compare the profiles of hepatic Th1, Th2, Th17 and Treg cells and investigate their potential relationships with hepatic fibrosis.

## Materials and methods

### Parasites and mice

To obtain *C*. *sinensis* metacercariae, fresh naturally *C*. *sinensis* metacercariae infected fish were collected from Tsitsihar City, Heilongjiang Province, People’s Republic of China (47.33°N, 123.97°E), then homogenized and digested with artificial gastric juice (0.7% pepsin in 1% HCl solution, pH 2.0) for overnight at 37°C after fishbone and fins were removed. Eventually, the metacercariae were isolated under a dissecting microscope and stored in Alsever’s solution at 4°C until required.

The six-week-old female C57BL/6 mice, BALB/c mice and FVB mice were all purchased from Shanghai Laboratory Animal Co., Ltd (SLAC, Shanghai, China) and kept in specific pathogen-free facility with a 12-h light and 12-h dark cycle of Xuzhou Medical University. Each strain of mice was divided into two groups randomly: one normal group (n = 5) and the other infected group (n = 5). All the mice were provided with sterile food and water *ad libitum*. The infected mice were orally gavaged with 45 metacercariae and monitored daily for diet and survival. The body conditions of all mice were observed and recorded daily during the whole procedure. No animals displayed suffering or distress, and moreover, no animals died prior to the experimental endpoints. Before being sacrificed on 28 days post infection, each mouse was in deep anesthesia conditions. All this study was conducted in strict accordance with the Guide for the Care and Use of Laboratory Animals of the Ministry of Health, China and complied by the Animal Ethics Committee of Xuzhou Medical University (No. SCXK<SU>2014–0003). All this efforts were aimed to minimize the suffering of these scientific animals.

### Histological examination

The left liver from each mouse were removed and fixed, embedded in 10% paraffin, and 3-μm serial sections were examined by H&E staining and Masson’s trichrome staining. All these specimens were eventually analyzed under an optical microscope and captured 6 photographs.

### Determination of hydroxyproline content

For detection the collagen, hydroxyproline, an amino acid characteristic of collagen, was determined. Approximately 50mg tissues were sampled from liver in each mouse and a commercially available kit which acquired from Jiancheng Institute of Biotechnology was used to determine the concentration of the hydroxyproline content. All the procedures making using of the reaction of oxidized hydroxyproline with 4-(Dimethylamino) benzaldehyde (DMAB) were performed in accordance with the manufacturer’s instructions.

### Isolation of hepatic leukocytes and detection of *C*. *sinensis* eggs in liver

For isolating the hepatic leukocytes, the modification of protocols previously described was used [27]. Briefly, the whole liver was isolated and homogenate totally when packaged in nylon membrane. Subsequently, 40% and 70% percoll density gradient was added into the sample carefully and then centrifuged at 2500 rpm for 25 min at room temperature. The lowest layer was used to examine the eggs microscopically and the middle layer which is the leukocytes was used for flow cytometry.

### Flow cytometry

For the analysis of Th1, Th2, Th17 subsets, approximately 10^6^ leukocytes isolated from liver were stimulated with 20 ng/ml PMA, 1 μg/ml ionomycin and 2 mmol/ml monensin (Sigma-Aldrich, St. Louis, MO, USA) for 4 h at 37°C in 5% CO_2_. For surface staining, the cells were collected and then incubated with anti-CD4-FITC for 30min. For intracellular staining, the cells were washed and fixed, permeabilized with Perm/Fix solution (eBioscience, San Diego, CA, USA) for 30 min and stained with anti- IFN-γ- Alexa Flour 488, anti-IL-4- APC and anti- IL-17A-PE (BD Biosciences) for 30 min for detecting Th1, Th2, Th17 cells, respectively. All samples were evaluated on flow cytometer (FACSCanto II; BD Biosciences, Franklin Lakes, NJ, USA) using Diva software (BD Biosciences).

For detection of Treg cells, the liver leukocytes were first surface incubated with anti-CD4-PerCP-Cyanine5.5 and anti-CD25-FITC for 30 min. Subsequently, Foxp3 staining buffer set (eBioscience, San Diego, CA, USA) were used to treat the cells and anti-Foxp3-APC were added for 1 hour. All samples were evaluated on flow cytometer (FACSCanto II; BD Biosciences, Franklin Lakes, NJ, USA) using Diva software (BD Biosciences). Samples were acquired and analyzed on a FACSCanto II flow cytometer.

### RNA isolation and quantitative RT-PCR

To detect the relative mRNA expression, hepatic total RNA of each mouse was extracted using TRIzol (Invitrogen, Carlsbad, CA) flowed by manufacturer's recommendations. The Nanodrop spectrophotometer (ThermoScientific, Wilmington, DE) was subsequently used to measure the RNA quality and concentration. cDNA was reverse transcribed from 1 μg total RNA per sample and then amplified using LightCycler480 real-time PCR System (Roche Diagnostics Inc). The primer sequences used to amplify the gene of interest were the following: β-actin (mβ-actin-F: 5'-TGGAATCCTGTGGCATCCATGAAAC-3'; mβ-actin -R: 5'-TAAAACGCAGCTCAGTAACAGTCCG-3'); IL-4 (mIL-4-F: 5'-AACGAGGTCACAGGAGAAGGGGT'; mIL-4-R: 5'-TCCAAGCAGGACAGAGAAAGCAT-3'); IL-5 (mIL-5-F: 5'-AGGCTTCCTGTCCCTACTCATAA-3'; mIL-5: 5'-TCTCTCCTCGCCACACTTCTCT-3'); IL-10 (mIL-10-F: 5'-GCTCTAGAAACACCTGCAGTGTGTATTGAGTCTGCTGGA-3'; mIL-10-R: 5'- GCTCTAGAAATTCGAATAAGATCCATTTATTCAAAATTAG-3'); TGF-β (mTGF-β-F: 5'-CACTGATACGCCTGAGTG-3'; mTGF-β-R: 5'-GTGAGCGCTGAATCGAAA-3'). Targeted mRNA expression levels were normalized to the housekeeping gene β-actin and then fold changes was calculated by comparison to the expression of the controls.

### Statistical analysis

All data were analyzed using SPSS16.0 and presented as mean ± standard error of the mean (SEM). One-way analysis of variance (ANOVA) was performed to analyze the statistical significance. Correlation studies were calculated using Pearson’s correlation. *P*-value<0.05 was deemed statistically significant.

## Results

### Gross observation and histopathological changes

**Fig 1:**
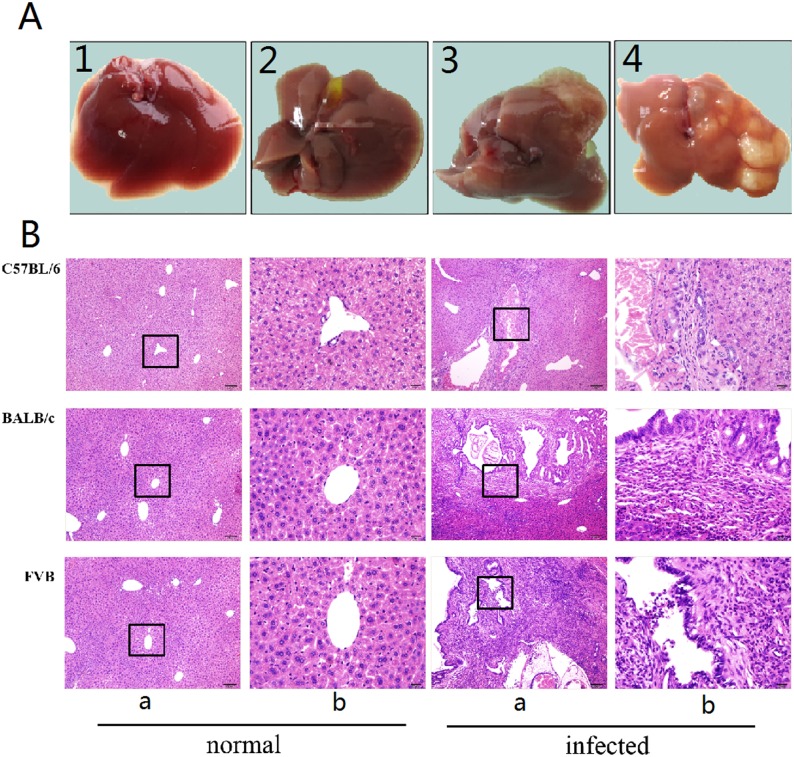
Gross observation and histopathological changes of livers in normal and *Clonorchis sinensis*-infected mice. Each strain of mice was randomly divided into normal group (n = 5) and *C*. *sinensis*-infected group (n = 5) which were orally infected with 45 metacercariae. All the mice were sacrificed on 28 days post infection. (A) Gross observation of livers. A-1, A-2, A-3 and A-4 represent the livers in normal mice, *C*. *sinensis*- infected C57BL/6 mice, *C*. *sinensis*- infected BALB/c mice and *C*. *sinensis*- infected FVB mice, respectively. (B) The histopathological changes of livers in C57BL/6, BALB/c and FVB mice were examined by H&E staining (×100).

Only 2 to 4 small white nodules were grossly found on the surface of the liver in each *C*. *sinensis* infected C57BL/6 mice. However, diffuse nodules were observed in the infected BALB/c mice, and more seriously, the liver in the *C*. *sinensis*-infected FVB mice showed swelling and several big whitish cysts. Adult or juvenile worms could be observed in these three strains of mice (Fig 1A). Interestingly, all the five infected C57BL/6 mice and only one infected BALB/c mice were egg negative.

As results showed in Fig 1B, although adult worms in the liver were observed in all strains, there were only moderate infiltration of inflammatory cells and fibrotic proliferation surrounding the proliferative bile ducts in the C57BL/6 mice, while more serious hepatic pathologies emerged in the other two strains, whose bile duct hyperplasia was more prominent and large area of fibrosis accompanied with wild inflammation changes even extended into the portal and periportal areas.

### FVB and BALB/c mice but not C57BL/6 mice showed marked biliary fibrosis

**Fig 2:**
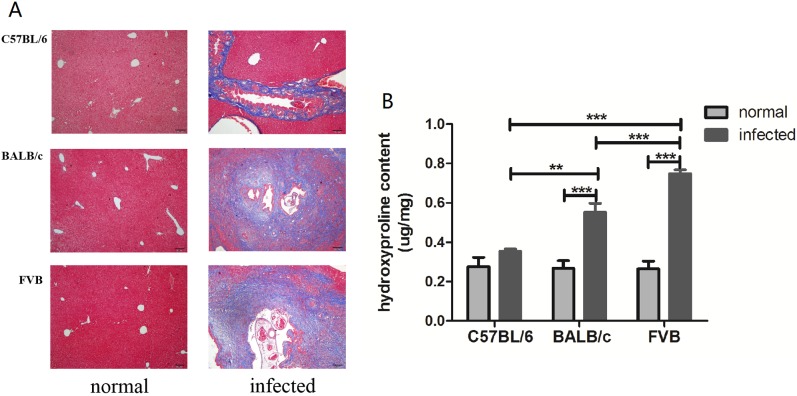
Determination of hepatic fibrosis in normal and *Clonorchis sinensis* infected mice. (A) Paraffin-embedded sections were stained by Masson’s trichrome and the blue area represents collagen deposition. (B) The concentration of the hydroxyproline content was tested in liver homogenate in three strains. Each value represents the mean±SEM of four or more independent experiments. ** = *P* <0.01, *** = *P* <0.001.

Masson’s trichrome staining was employed to further assess the fibrotic changes in three strains. *C*.*sinensis*-infected C57BL/6 mice showed a moderate trend of ECM and collagen fibers were only deposited around portal areas while such changes were more extensive in FVB and BALB/c mice (Fig 2A). In addition, as Fig 2B showed, compared with *C*. *sinensis*-infected C57BL/6 mice, a higher level of hydroxyproline contents were observed in FVB mice (*P*<0.001, Fig 2B) and BALB/c mice (*P*<0.01, Fig 2B). Taken together, *C*. *sinensis*-infected FVB mice showed the most intensive fibrosis, followed by *C*. *sinensis*-infected BALB/c and C57BL/6 mice.

### FVB and BALB/c mice have increased hepatic Th2 and Treg subsets

**Fig 3:**
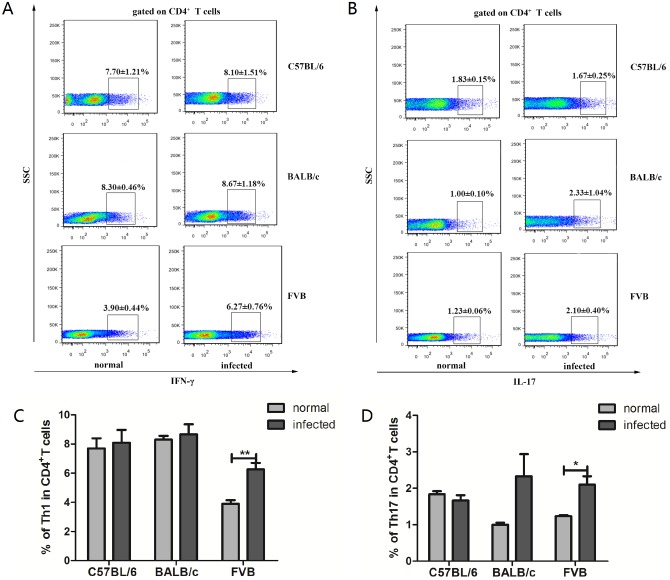
The percentages of hepatic Th1 and Th17 cells in normal and *Clonorchis sinensis*-infected mice. Data shown are gated on CD4^+^T cells. Numbers represent the ratios of Th1 (A) and Th17 (B) in CD4^+^T cells. (C) & (D) indicated the changes of Th1 and Th17 in three strains of mice, respectively. Data are mean±SEM of four or more independent experiments. * = *P* <0.05, ** = *P* <0.01, *** = *P* <0.001.

**Fig 4:**
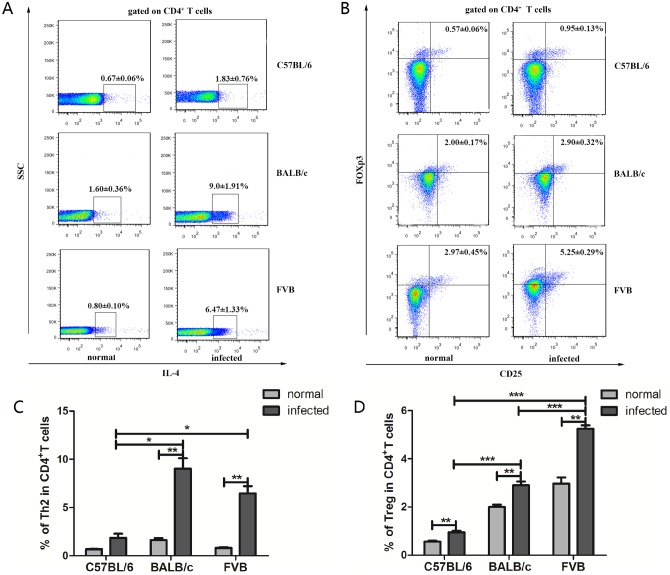
The percentages of hepatic Th2 and Treg cells in normal and *Clonorchis sinensis*-infected mice. Data shown are gated on CD4^+^T cells. Numbers represent the ratios of Th2 (A) and Treg (B) in CD4^+^T cells. (C) &(D) indicated the changes of Th2 and Tregs in three strains of mice, respectively. Data are mean±SEM of four or more independent experiments. * = *P* <0.05, ** = *P* <0.01, *** = *P* <0.001.

As results showed in Fig 3, the percentages of hepatic Th1 (*P*<0.01, Fig 3C) and Th17 (*P*<0.05, Fig 3D) were significantly higher in *C*. *sinensis*-infected FVB than those non-infected FVB mice and there were no any differences for these two subsets in the other two strains (*P*>0.05, Fig 3C and 3D). Furthermore, any differences of the Th1 subset and Th17 subset were found among the three strains (*P*>0.05). However, compared with the each normal group accordingly, either Th2 or Treg cells was dramatically increased in the *C*. *sinensis*-infected BALB/c group or FVB group (*P*<0.01, Fig 4). Moreover, Th2 subset was significantly higher in *C*. *sinensis*-infected FVB and BALB/c mice than those in *C*. *sinensis*-infected C57BL/6 mice (*P*<0.05, Fig 4C). Similar trend was shown about Treg cells (*P*<0.001, Fig 4D).

**Fig 5:**
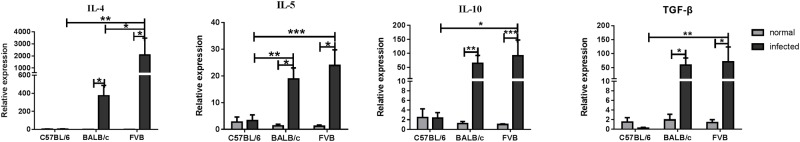
The levels of mRNA of IL-4, IL-5, IL-10 and TGF-β cytokines in the liver of normal and *Clonorchis sinensis*-infected mice. mRNA of IL-4(A), IL-5(B), IL-10(C) and TGF-β(D) cytokines in the liver were determined by quantitative RT-PCR. All data are mean±SEM of four or more independent experiments. * = *P* <0.05, ** = *P* <0.01, *** = *P* <0.001.

To ascertain this FACS data, we determined the relative expression of Th2 and Treg related cytokines (IL-4, IL-5, IL-10 and TGF-β) in the liver of non-infection and *C*. *sinensis* infection mice by the quantitative RT-PCR. The results showed that the transcripts of IL-4, IL-5, IL-10 and TGF-β in the liver of *C*. *sinensis* infected mice were all significantly higher than those non-infected mice with same background (Fig 5). For different strains of *C*. *sinensis*-infected mice, consistent with the data of Flow FACS, the results showed that the levels of these cytokines in *C*. *sinensis*-infected BALB/c mice and FVB mice were considerable increased, compared with C57BL/6 mice infected by *C*. *sinensis* (Fig 5).

### The increased hepatic Th2 and Treg subsets were positively correlated with liver fibrosis in three strains of mice

**Fig 6:**
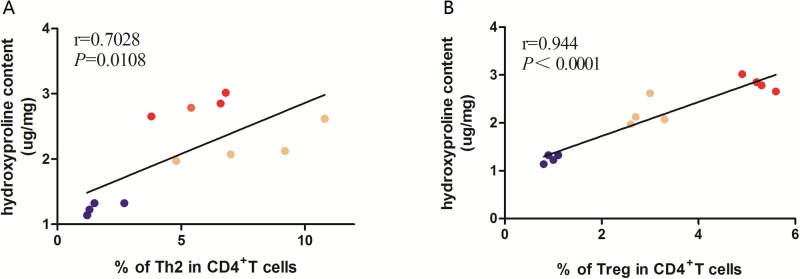
Correlations between Th2, Treg and hydroxyproline concentrations during *Clonorchis sinensis* infection within different strains of mice. Pearson’s correlation was used to analyze the associations between these two CD4^+^T cell subsets and hydroxyproline contents. The blue, yellow and red dots represent C57BL/6 infected mice, BALB/c infected mice and FVB infected mice, respectively.

Th2 and Treg cells are probably involved in liver fibrosis caused by the infection of *C*. *sinensis* [26, 28]. To investigate the potential roles of the increased Th2 and Treg subsets in regulation of fibrogenesis in different strains of mice, the correlation analysis was used to analyze the associations between these two CD4^+^T cell subsets and hydroxyproline contents. As the results showed in Fig 6A, a significantly positive correlation was found between Th2 subset and hydroxyproline contents (r = 0.7028, *P*<0.01) and a similar trend was also observed between Treg subset and hydroxyproline contents (r = 0.944, *P*<0.0001, Fig 6B).

## Discussion

As Uddin has compared the susceptibility in 6 strains (ICR, BALB/c, C57BL/6, DDY, CBA/N, and C3H/HeN) of mice, there are apparent differences in worm recovery, histopathological changes, and immunoglobulin, as well as cytokines levels to *C*. *sinensis* infection among these different strains [29]. However, the mechanisms underlying of the host-parasite interactions contributing to these differences are not fully elucidated. It is well characterized that the liver injuries might be induced by *C*. *sinensis* with the prolonged mechanical irritation, nitric oxide formation, oxidative stress and intrinsic nitrosation [30–32]. But the pattern of hepatic CD4^+^T cell subsets and their possible relationships with liver histopathologic changes in different strains of mice are still unclear.

Previous studies demonstrated that H2 haplotypes have a dramatic influence on the lesions and immune responses caused by infective and autoimmunity diseases [33–35]. It also has been previously reported that, in terms of *Trypanosoma cruzi* and *Echinococcus granulosus* infection, BALB/c (H-2d) was verified to be a more susceptible strain than C57BL/6 (H-2b) mice. Additionally, more extensive Th2 responses and more IL-4 cytokine production were examined in BALB/c mice [36, 37]. Interestingly, FVB (H-2q) mice possess relative susceptibility to *C*. *sinensis* infection than BALB/c mice but are resistant against infection with murine filariasis, and *Nippostrongylus brasiliensis* [38–40]. In this study, FVB, BALB/c and C57BL/6 were employed and orally fed with 45 metacercaria. Adult worms were seen in the livers of all the three strains at 28 days post-infection, which implies that such infection models were established successfully. Our histopathological results showed that, in contrast to C57BL/6 mice, the severity of inflammatory cells recruitment, bile ducts proliferation and collagen depositions were all significantly increased in FVB and BALB/c mice. Furthermore, these changes were the most severe in FVB mice. Overall, our findings were consistent with the previous studies [29, 38].

Increasing evidences support the concept that the cytokines secreted by Th1 cells increased the abilities to eliminate parasites [39]. Therefore, *C*. *sinensis* adult worms could successfully survive in the hosts that who showed the depressed Th1 responses. But Th2 cytokines such as IL-4, IL-10 and IL-13 are associated with the aggravated pathological injuries and our previous study showed that the imbalanced Treg/Th17 may play a role in the formation of liver fibrosis [26, 41]. There is a controversy concerning the roles of Treg cells playing in the context of experimentally induced fibrosis [42]. For instance, the depletion of Tregs in mice has been verified to inhibit the progression of lung fibrosis. However, in an experimental cardiac fibrosis, Tregs played a protective role in tissue fibrosis [43, 44]. Moosbrugger-Martinz also reported that the up-regulation of pathogenic Tregs in atopic dermatitis is able to produce Th2 cytokines and to promote the disease instead of alleviating symptoms [45]. The effects of CD4^+^CD25^hi^Foxp3^+^ Tregs are altered in the environment stimulated by bleomycin with the IL-2 complex, which ultimately leads to heightened pulmonary fibroplasia by activating dominant type 2 immune responses [46]. It is corroborated that Th2 immune responses which are responsible for the resistance of hosts to the helminthic infection could activate TGF-β1 via producing IL-4 and IL-13 and TGF-β signaling pathway, and the latter in turn acts as a prominent mediator in fibrosis [41]. Our result showed that the hepatic Th2 and Treg cells and related cytokines IL-4, IL-5, IL-10 and TGF-β were dramatically increased in the BALB/c and FVB infected groups compared with the C57BL/6 infected group. The positive correlation between the increased Th2 subset and Treg subset with hydroxyproline contents suggest that the augmentations of Th2 and Treg could be a medical targets as they may play a potential role inducing liver fibrosis in different strains of mice, but the underlying mechanism is unclear and warrant to be addressed in further studies.

### Conclusions

The present study showed that a diverse change in profile of the hepatic CD4^+^T cell subsets including Th1, Th2, Th17 and Treg in *C*. *sinensis* infected C57BL/6, BALB/c and FVB mice. This result implied that Th2 and Treg cell subsets were likely to be important in the formation of hepatic fibrosis, which may provide fundamental information for further studying the mechanism of CD4^+^T cell subsets in clonorchiasis.
